# Quantitative resistance differences between and within natural populations of *Solanum chilense* against the oomycete pathogen *Phytophthora infestans*

**DOI:** 10.1002/ece3.7610

**Published:** 2021-05-11

**Authors:** Parvinderdeep S. Kahlon, Melissa Verin, Ralph Hückelhoven, Remco Stam

**Affiliations:** ^1^ TUM School of Life Sciences Technical University of Munich Freising Germany

**Keywords:** natural populations, *Phytophthora infestans*, quantitative resistance, *Solanum chilense*

## Abstract

The wild tomato species *Solanum chilense* is divided into geographically and genetically distinct populations that show signs of defense gene selection and differential phenotypes when challenged with several phytopathogens, including the oomycete causal agent of late blight *Phytophthora infestans*. To better understand the phenotypic diversity of this disease resistance in *S*. *chilense* and to assess the effect of plant genotype versus pathogen isolate, respectively, we evaluated infection frequency in a systematic approach and with large sample sizes. We studied 85 genetically distinct individuals representing nine geographically separated populations of *S*. *chilense*. This showed that differences in quantitative resistance can be observed between but also within populations at the level of individual plants. Our data also did not reveal complete immunity in any of the genotypes. We further evaluated the resistance of a subset of the plants against *P*. *infestans* isolates with diverse virulence properties. This confirmed that the relative differences in resistance phenotypes between individuals were mainly determined by the plant genotype under consideration with modest effects of pathogen isolate used in the study. Thus, our report suggests that the observed quantitative resistance against *P*. *infestans* in natural populations of a wild tomato species *S*. *chilense* is the result of basal defense responses that depend on the host genotype and are pathogen isolate‐unspecific.

## Graphical abstract

Populations of the wild tomato species *Solanum chilense* show large variation in quantitative resistance properties against the oomycete pathogen *Phytopjthora infestans* between and within populations. This variation is largely defined by the plant genotype and not by the pathogen genotype and thus likely represents a form of variation in basal immune responses.

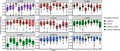

## 1 INTRODUCTION

Plant immunity can be observed in a qualitative manner, in which plants are either completely resistant or susceptible, or in a quantitative way, in which certain plants of the same species are more resistant and others are less resistant. Such intraspecific quantitative variation in resistance was formally introduced as horizontal resistance in the field of phytopathology by Vanderplank in 1963, and he further extended his concept in 1968. By his definition, horizontal resistance is a product of multiple underlying genes, that all have minor effects on the amount of resistance observed. This may explain why quantitative resistance is observed to be pathogen genotype independent in many cases. This is in contrast to vertical resistance, also called qualitative resistance, that depends on a single major resistance (*R*) gene and is pathogen genotype dependent. Here, the product of specific *R* gene recognizes a specific pathogenic protein to deliver the resistance. Thus, plants with quantitative or horizontal resistance show a range of intermediate infection phenotypes but no complete resistance. Today the theories and concepts proposed by Vanderplank are well integrated in plant pathology and in use by plant disease epidemiologist in the field (Coutinho et al., 2020).

For natural populations, knowledge on quantitative resistances is limited, when compared to agriculture. However, a number of species have been reported to show quantitative resistance in their natural populations against several pathogens as highlighted in a review by Laine et al. (2011). Burdon (1980) was one of the first to show in detail the differences in resistance to the fungal pathogen *Cymadothea trifolii* and *Pseudopeziza trifolii* in a population of white clover, *Trifolium* *repens*, with a significant difference observed among the least and most resistant genotypes. Another notable example is a study on two populations of *Senecio vulgaris*. The species was shown to have up to 10 different resistance phenotypes against the powdery mildew fungus *Erysiphe fischeri* within an area of 1 m^2^ of the population (Bevan et al., 1993). Some recent reports also highlight interesting aspects underlying the quantitative resistance observed. A study on six natural genotypes of bittersweet, *Solanum dulcamara*, showed differences in tolerance and resistance against *P*. *infestans* and interestingly highlights a mechanism of overcompensation in some genotypes with increase in shoot length and number of flowers in plants with higher disease levels (Masini et al., 2019). Ultimately, these studies highlight host genotype‐specific differences in resistance to biotic stress in wild plant species and show the potential of natural populations to be a valuable resource to explore and exploit quantitative resistance for evolutionary and economical aspects.

Here, we investigate phenotypic variation in resistance of the wild tomato species *Solanum chilense*. The natural habitat of *S*. *chilense* is southern Peru and northern Chile. The demography of this species can be described by four clearly defined genotype groups based on four geographical regions: one northern, one central, and two southern groups (Böndel et al., 2015). The regions are geographically separated from each other, and thus, all have their own specific climate. As a result, the populations growing in these four regions are genetically different and abiotic stress adaptations have been reported for example for genes involved in cold‐ and drought‐stress responses (Böndel et al., 2018; Fischer et al., 2013; Nosenko et al., 2016). An advantage of working with *S*. *chilense* is that it is perennial. Hence, each plant represents a unique genotype from a distinct population but can be kept in the greenhouse and regularly cut back to maintain identical genetic material for years and comparable experiments.

The species has also been established as a genetic model for studies on plant–pathogen interactions. Some accessions of the species possess resistance genes against the fungal pathogen *Verticillium dahlia* or to tomato mottle virus (Griffiths & Scott, 2001; Tabaeizadeh et al., 1999). Genes of the *Pto* resistance pathway, conferring resistance to *Pseudomonas* sp., are shown to be under balancing selection (Rose et al., 2011). Stam et al. (2019) previously showed that also *R* genes of the nucleotide‐binding site, leucine‐rich repeat receptor (NLR) gene family, show signatures of adaptation across the species. Using targeted resequencing of these important receptor genes, they showed that it depends on the population whether *NLRs* are under positive or balancing selection and that only a few *NLRs* show consistent positive or balancing selection signatures across the species. This led to the conclusion that indeed *S*. *chilense* is a highly variable host species, but that some adaptations, also in pathogen defense‐associated genes, depend on or even drive colonization of new habitats (Stam et al., 2019). Most recently, we showed that genetic diversity in the species also has immediate effect on the observed resistance phenotypes of the host populations. Two specific *Cladosporium* *fulvum* resistance gene‐dependent responses show presence–absence variations between and within populations of the central geographical group of *S*. *chilense* (Kahlon et al., 2020). Interestingly, in this study, we also found complete loss of major *R* gene‐mediated resistance responses in the southern part of the species range, which we hypothesize to be related to the extreme environments and probably absence of the pathogen in these regions.

The presence and absence of these *R* genes results in qualitative differences in resistance. However, as mentioned above, many interactions in nature are of quantitative nature. Populations from geographically distinct locations in the *S*. *chilense* species range show such quantitative variation in the resistance against three common filamentous pathogens of tomatoes, *P*. *infestans*, *Alternaria* *solani*, and a *Fusarium* species (Stam et al., 2017). The resistances against these three pathogens do not follow geographic patterns, but instead show a mosaic pattern of resistance quantities, which led to the conclusion that local pathogen pressures and separation of individual niches likely differ even within geographical regions.

The oomycete *P*. *infestans* belongs to the Peronosporaceae family and has large economical relevance (Kamoun et al., 2015). It was the cause of the Irish potato famine, and it causes significant losses as late blight pathogen on tomatoes and other *Solanaceae* species (Kaushal et al., 2020). It was one of the first oomycetes to have its genome sequenced (Haas, 2009), making it one of the best studied oomycetes at the genomic level. *P*. *infestans* contains more than 500 genes coding for so‐called effectors, which are small specific proteins with recognizable amino acid domains that can contribute to the virulence of the pathogen (e.g., Bos et al., 2010). It is also known that the effector repertoire varies between isolates, with complete presence–absence variations as well as point mutations occurring in important effector genes (Thilliez et al., 2019). *P*. *infestans* occurs globally, with large variation between field populations: some being highly diverse, some being clonal, and some following sexual and some asexual reproduction mode (Fry et al., 2015). The origin of *P*. *infestans* has been either in the Andes (Gómez‐Alpizar et al., 2007; Martin et al., 2014) or in the Toluca Valley in Mexico (Goss et al., 2014). *P*. *infestans* has likely been present in areas home to wild tomato species for a long time. A recent publication confirms that *P*. *infestans* can regularly be detected on wild potato and several tomato populations, growing in close proximity to cultivated potato fields in the Peruvian Andes (Lindqvist‐Kreuze et al., 2020). Where cultivated potato and tomato crops are decimated when infected with these *P*. *infestans* isolates, these wild plants show often limited infection symptoms. Infection lesions appear to be smaller and do not spread over the whole plant (Lindqvist‐Kreuze et al., 2020), suggesting a high level of quantitative resistance to be present in the wild plant populations.

To evaluate above hypothesized presence of quantitative resistance from former mentioned studies, in this study, we looked in detail at the phenotypic variation observed in natural populations of *S*. *chilense*, tested the quantitative resistance against various isolates of *P*. *infestans*, and elucidated the role of plant genotype and the pathogen isolate in determining quantitative resistance.

## 2 MATERIALS AND METHODS

### 2.1 Plants material and maintenance

Seeds of nine accessions/populations of *S*. *chilense* (LA1958, LA1963, LA2747, LA2932, LA3111, LA3786, LA4107, LA4117A, and LA4330) were obtained from the C. M. Rick Tomato Genetics Resource Center of the University of California, Davis (TGRC UC‐Davis, http://tgrc.ucdavis.edu/). Each population represents a random collection of seeds from a wild population from geographically distinct locations (Figure S1) and is propagated by TGRC in a way to maintain maximum genetic diversity. Thus, each of the populations used is genetically distinct. The species is highly heterozygous so each individual plant within a population is also genetically unique and can be considered a different genotype. For each population, 9–10 plants were grown in controlled greenhouse conditions (16h light and 18–20°C temperature). Mature plants of at least one‐year‐old were used in the study and were maintained throughout the study by bi‐weekly cutting. The plants are perennial and can be maintained for a longer time and providing the benefit of allowing repeated testing of the same plants.

### 2.2 Pathogen material and maintenance

*Phytophthora infestans* isolates EC1, NL88069, 06_3928A, and T30‐4 were obtained from Wageningen University, Pi100, Pi078, Pi054, were obtained from INRAE Avignon. We used these *P*. *infestans* isolates as they have been reported to have differences in effector diversity, originating from different geographical locations and different hosts shown in Table S1 (Caromel et al., 2015; Thilliez et al., 2019). All the isolates were maintained on long‐term maintenance medium, Rye A Agar (Stam, 2016a), and were subcultured on sporulation medium, Rye B Agar (Stam, 2016b), before experiments and incubated 9 days in the dark at 16°C and 1 day in the light at room temperature. The sporangia were scratched from the plates after flooding with water, with the help of a pipette tip and stored at 4°C for 1–2 hr prior to infection assays. The final concentration used for the infection assays and pathogen phenotype assays was 3,000 sporangia/ml.

### 2.3 Detached leaf infection assays with isolate Pi100

The infection assays were performed on 85 plants representing nine populations of *S*. *chilense* using a drop inoculation method with sporangia of *P*. *infestans* isolate Pi100 on the detached leaf as described in Stam, Silva‐Arias, et al. (2019). Leaves on our glasshouse plants differed in age and size. Therefore, the assays were performed in three biological replicates, each several weeks apart. For each biological replicate, seven to eight randomly selected leaves were harvested per plant from each of the mother plants (making up to 24 technical replicates per plant) and mother plants were cut back between experiments. Due to large number of plants and technical replicates, experiments were performed in two sets; in the first set, we tested five populations (LA1963, LA2932, LA3111, LA3786, and LA4330), and in the second set, we tested four populations (LA1958, LA2747, LA4107, and LA4117A). Population LA1963 was included in the second set as an internal calibrator to test potential effects of different experimental dates (Table S2).

To avoid influence of epiphytes in our infection outcome, we first evaluated presence of epiphytes in our mother plants in 10 randomly selected leaves. Each leaf was cut into four explants and transferred on four plates with commonly used culture medium for microorganism propagation, Luria–Bertani agar, V8 agar, 0.25 potato dextrose agar, and Spezieller Nahrtoffarmer agar, compositions shown in Table S3, and we used 70% surface sterilization to check the effect on reducing the commonly present epiphytes. We visually evaluated the presence of contaminants 7 days postexplant transfer (Figure S2). As surface sterilization proved to be an effective method to avoid influence of fungal epiphytic contaminants, we performed surface sterilization of leaves with 70% ethanol for further assays, by dipping the leaves in the solution briefly, rinsing with water and gently dabbing with a dry tissue to remove the excessive water. The leaves were then placed in plastic boxes (50 × 32.5 × 6.5 cm) containing bedding of two layers of wet tissue paper as shown in schematic in Figure S1, with the abaxial site facing upward. Drop inoculation with 5 µl volume of liquid containing 3,000 sporangia/ml was performed on the individual leaflets of each leaf (up to 18 leaflets per leaf), and boxes were kept in the dark at 18–20°C. We observed still minor amount of bacterial contamination after surface sterilization (Figure S2). To allow ruling out its influence in the outcome of our infection assays, we simultaneously performed control inoculations with water on four leaves for each plant in each biological replicate and we kept the plants out of the analysis that showed clear presence of the contaminants on either control or inoculated leaves (Figure S3). Seven days postinoculation, the infection frequency was recorded per individual leaf by calculating the ratio of the number of leaflets showing mycelia growth divided by the total number of leaflets inoculated. A leaflet was counted as infected when we observed lesions that were larger in size than the originally placed droplet. Every infection event has a certain amount of stochasticity, as spore germination rates are not uniform, even under optimal conditions (Minogue & Fry, 1981). To reduce the variance caused by this effect, we performed a minimum of 1,588 drop inoculations per population. This number accumulates from a total of three independent experiments with inoculation of all individual leaflets of each seven to eight leaves from nine to ten individual plants per population.

### 2.4 Phenotyping different *P*. *infestans* isolates on culture medium and plants

Differences in mycelial growth type of seven isolates were first confirmed on Rye A agar plates when the mycelial fully covered the plates after agar plug transfer (Figure S4). To investigate the growth rate of these isolates, drop inoculations of the same sporangia solution used for the infections were done on Rye B Agar culture medium and incubated at 18–20°C in the dark. Phenotyping was performed with up to five technical replicates and three biological replicates per strain. Each biological replicate was performed at least 10 days apart, with freshly propagated starter cultures. The diameter of the mycelia growth (in cm) was taken 10 days postinoculation. Additional infection assays were performed on the population LA3111 (nine plants), which is a population from the central region of the species demography. We choose this population due to availability of a reference genome from this population (Stam, et al., 2019). The same drop inoculation method was performed with 3,000 sporangia/ml of seven isolates of *P*. *infestans* (Pi100, Pi078, Pi054, EC1, NL88069, 06_3928A, and T30‐4) on the detached leaves. The infection frequency was obtained from individual plants, using the same methodology as described above.

### 2.5 Statistical analysis and data representation

Analysis of variance was performed using the function aov() in R software, version 3.4.4 (R Core Team, 2017), with post hoc Tukey test function TukeyHSD(), from the package {stats}, and *p*‐value was considered significant when lower than 0.05. Generalized linear mixed models (GLMM) were generated for the population LA3111 infection assay with multiple *P*. *infestans* isolates to evaluate the effect of plant genotype and pathogen isolate on the infection outcome using glmer() from the package {lme4} (Bates et al., 2015), as shown in Stam, Silva‐Arias, et al. (2019), and we used the binomial variable (y) which consisted of number of successful and unsuccessful infection events per leaf (yes and no infection event counts), and experimental date was used as a random effect in the both models. Figures were made using the R package {ggplot2} (wickham 2016).

## 3 RESULTS

### 3.1 Quantitative resistance differences between populations are independent of the pathogen isolate

To know whether populations of *S*. *chilense* possess quantitative resistance differences upon inoculation with a very aggressive *P*. *infestans* isolate Pi100, originating from *Solanum lycopersicum* in Europe, we performed drop inoculations on the detached leaves.

**FIGURE 1:**
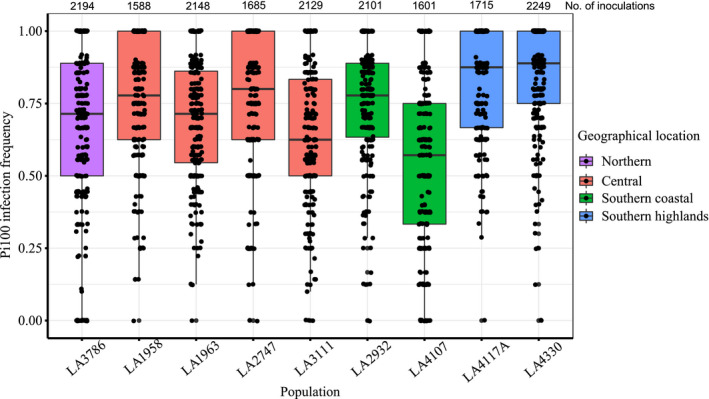
Infection frequency in different populations of *S*. *chilense* upon inoculation with *P*. *infestans* isolate Pi100. The box plots show the median of the infection frequency of a leaf which is the ratio of infected leaflets over total inoculated leaflets. Each population consisted of 9–10 plants. The assay was performed on three separate dates, each time with seven to eight leaves for each individual plant. Each data point indicates the infection frequency of an individual leaf obtained from inoculations of up to 18 leaflets per leaf. The *y*‐axis shows infection frequency ranging from 0 (no infected leaflets on a leaf) to 1 (all leaflets show infection). The colors represent the geographic regions of the population

We observed differences in quantitative resistance in the different populations upon infection with *P*. *infestans* isolate Pi100 (Figure 1). LA4107 was most resistant population and LA4330 most susceptible. We evaluated the effect of date of experiment using ANOVA with post hoc Tukey honest significant difference test and found no significant differences among the different dates of experiments (Table S2).

In our study, all plants were infected. ANOVA with post hoc Tukey honest significant difference tests showed significant differences between populations in 23 out of 36 pairwise comparisons (Table S4). Previously, Stam, Silva‐Arias, et al. (2019) showed similar quantitative resistance differences when infected with *P*. *infestans* isolate EC1. We calculated the Pearson's correlation coefficient between the overlapping population's mean score of infection frequency from data on EC1 from the previous study (Stam, Silva‐Arias, et al., 2019) and our infection frequency data on Pi100 from this study. This coefficient of 0.724162 indicates a positive correlation between the two data sets, which means that the *S*. *chilense* populations behave similarly to the two isolates. Due to a limited sample size (*n* = 6 overlapping populations), we cannot reasonably judge the significance of this correlation. We further had up to six nonoverlapping plants per population between the experiments accounting for additional variance within our samples. However, these results suggest that the variation in the infection frequency is the result of genetic differences in the host plants.

### 3.2 Individual plant genotypes appear to drive resistance against single *P*. *infestans* isolate

To test whether the observed variance in the infection frequencies (Figure 1) is intrinsic to the assays or to the population as a whole or whether significant differences between the individual plants in the population contribute to the large variance.

**FIGURE 2:**
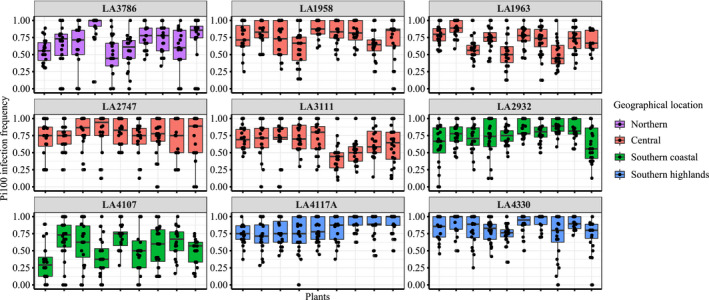
Evaluation of infection frequency in individual plants from different populations of *S*. *chilense*. Each individual facet shows different populations (as tested in Figure 1), and each box plot shows a single plant tested for infection phenotype. Experiments were performed on three separate dates (with an exception for one plant only on two dates), each time with eight leaves per plant (with an exception in two plants with seven leaves per plant). *Y*‐axis represents infection frequency, and *x*‐axis represents different plants; color shows the geographical region of the population (as in Figure 1)

To evaluate the infection frequency within the population, we separated the data of the drop inoculation on all detached leaves (Figure 1) into infection frequency for each of the individual plants (Figure 2). We observed high variance in the successful infection frequency in individual plants within the population. In the majority of cases (68% of the plants), this variance was smaller than the variance observed in infection frequency for the population as a whole (Tables 1 and S5). This indicates that the differences in successful infection of *P*. *infestans* can at least partly be attributed to genetic differences within the host populations; however, other factors beyond our control cannot be disregarded in this outcome (i.e., physiological differences between individual leaf explants from perennial plants).

**TABLE 1:** Variance observed in infection frequency in *S*. *chilense* populations against *P*. *infestans*. Table highlights variance of overall populations and number of plants which show high and low variance when compared to overall population variance

Population	Variance in infection frequency	Number of plants with higher variance than overall population	Number of plants with lower variance than overall population	Number of plants tested
LA3786	0.0815094	4	6	10
LA1963	0.044541	2	8	10
LA1958	0.047965	3	6	9
LA2747	0.0615848	4	5	9
LA3111	0.0605912	3	6	9
LA2932	0.0441113	3	7	10
LA4107	0.0828558	4	5	9
LA4330	0.0375442	3	7	10
LA4117A	0.0367892	2	7	9

To test whether the observed differences in infection frequency for each of the plants were statistically significant, we performed an ANOVA, with post hoc Tukey honest significant difference test for all plant–plant comparisons within a population (Table S6). For each population, upto 45 pairwise comparisons were evaluated (amounting to 360 comparisons), out of which 68 were significantly different at *p* < .05. The number of significantly different comparisons varied between the populations. Most frequent significant differences were found within the populations LA1963 (18) followed by LA3111 (8), LA3786 (8), LA2932 (8), LA4107 (7), LA4117A (7), LA4330 (7), and LA1958 (5), whereas LA2747 showed no significant difference in the plants’ pairwise comparison. This confirmed that the plant genotypes within populations showed different resistance phenotypes against a single isolate of *P*. *infestans*.

### 3.3 Growth rate of different isolates of *P*. *infestans* on culture medium is significantly different

**FIGURE 3:**
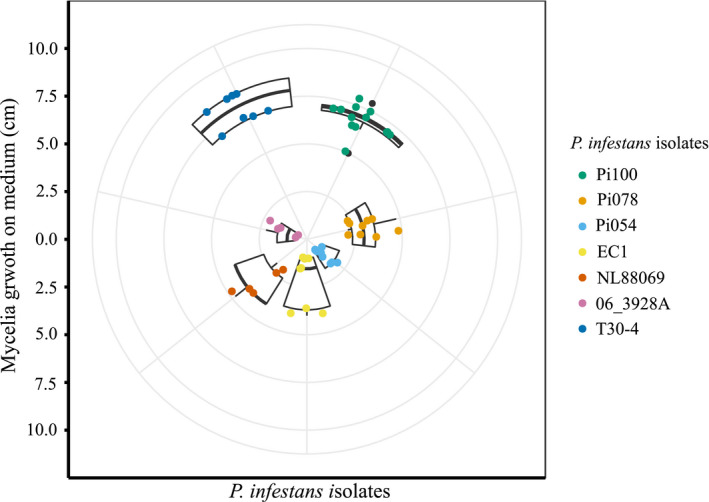
Growth of different isolates of *P*. *infestans* on culture medium. The radial plot shows the outgrowth of mycelia (in cm) of different isolates of *P*. *infestans* 10 days postdrop inoculation on Rye B Agar medium. Different colors indicate different isolates of *P*. *infestans*

To understand whether resistance was isolate‐specific, we selected seven diverse isolates of *P*. *infestans*. To first judge general differences of these seven isolates apart from the host plant, we evaluated their growth rate in vitro. To measure phenotypic growth differences, we performed drop inoculation of sporangia solution (3000/ml) of the *P*. *infestans* isolates on the culture medium Rye B Agar B (Figure 3) and measured the radial outgrowth. The best growing isolate was T30‐4, and the slowest growing was Pi054. The outgrowth of the isolates was significantly different for 66.67% of the pairwise comparisons confirming growth differences of these isolates on the culture medium (14 pairwise comparisons out of 21 pairwise comparisons—ANOVA, with post hoc Tukey honest significant difference test; Table S7).

### 3.4 Infection with seven different isolates of *P*. *infestans* supports host genotype dependency

**FIGURE 4:**
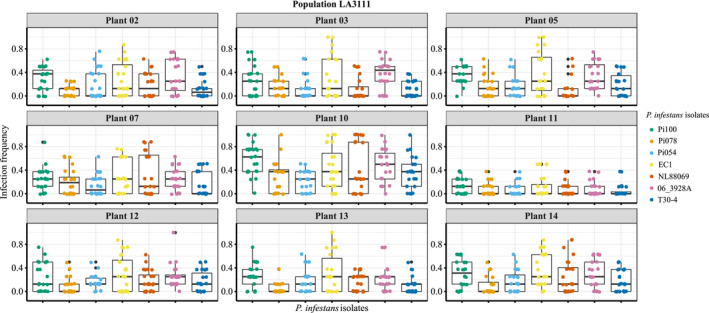
Infection frequency of seven different isolates of *P*. *infestans* on plants within *S*. *chilense* population LA3111. Each facet shows an individual plant of the population LA3111, the box plot represents the infection fraction of plants when inoculated with seven isolates of *P*. *infestans* depicted in different colors. *Y*‐axis represents infection frequency (0–1 as in Figures 1 and 2), and *x*‐axis represents different *P*. *infestans* isolates

Knowing that the selected isolates are genetically and phenotypically different, we further evaluated infection frequency of the seven isolates of *P*. *infestans* on the detached leaves from the nine individual plants of the central population LA3111. We observed that the plant showing the highest resistance (i.e., the lowest infection frequency), upon inoculation with Pi100 (Plant 11), is always the most resistant irrespective of the *P*. *infestans* isolate used. Similarly, the plant showing the lowest resistance (i.e., the highest infection frequency) to Pi100 (Plant 10) is always the least resistant irrespective of the isolate used (Figure 4).

To test if we observe an effect of the pathogen isolate on the infection frequency on plant genotypes, we performed ANOVA, with post hoc Tukey honest significant difference tests and found 10.05% of the pairwise comparisons to be significantly different (Table S8). To evaluate the differences in infection frequency correlation to growth rate of the *P*. *infestans* isolates, we compared the mean growth of *P*. *infestans* isolates on the plates with the mean of infection frequency of these isolates on the plants. We saw no correlation upon evaluation with a Pearson's correlation test (coefficient of −0.064, *n* = 7).

Finally, we tested the effect of the plant genotype and the pathogen isolate on the infection frequency via multiway ANOVA. We found both, the effect of the plant genotype and the pathogen isolate to be significant but independent of each other (Table 2). This supports that no clear effect exists of the interaction between specific plant genotypes and specific isolates, as would be expected for qualitative, race‐specific resistance. Rather, we observe independent effects on our infection frequency outcomes. We performed a GLMM model using the inoculation date as random effect and as expected, the additive model (Plant + Pathogen) provided the best fit (AIC and BIC criteria), while the plant genotype explained most of the differences in infection frequency (Table 3). Overall, we observed that *P*. *infestans* growth rate on medium does not correlate to infection success but we did observe plant genotype and pathogen isolate contribution to the outcome of the infection with plant genotype having the larger effect.

**TABLE 2:** Multiway ANOVA test outcome to evaluate effect of interaction of plant genotype of central population LA3111 of *S*. *chilense* and *P*. *infestans* isolate on infection frequency

Component	*df*	*F*‐value	*p*‐Value
Plant	8	21.971	<2e‐16
Isolate	6	22.794	<2e‐16
Plant:Isolate	48	1.179	0.19

**TABLE 3:** GLMM showing plant genotype and pathogen isolate effect on infection frequency observed in the central population LA3111 of *S*. *chilense* upon inoculation with seven isolates of *P*. *infestans*. The lower AIC and BIC predicts a better fitting model. To construct the models, we used the binomial variable (y) which is infection outcome, random effect considered in all models was date of the infection

Model	AIC	BIC
y ~ Plant	4,881.3	4,934.0
y ~ Isolate	4,981.0	5,023.1
y ~ Plant + Pathogen	4,548.5	4,632.8

## 4 DISCUSSION

*Solanum chilense* is known for its resistance against various pathogens (Tabaeizadeh et al., 1999; Griffiths & Scott, 2001; Ji et al., 2007; Verlaan et al., 2013). It has also been studied extensively in relation to habitat adaptation (Böndel et al., 2018; Fischer et al., 2013; Nosenko et al., 2016; Xia et al., 2010) and as a model for the evolution of pathogen defense mechanisms in plants (Kahlon et al., 2020; Stam, et al., 2019; Stam, et al., 2019). Previously, *S*. *chilense* populations from different geographical regions were shown to express different levels of resistance against three filamentous tomato pathogens (Stam, Silva‐Arias, et al., 2019). In this report, we further characterize the resistance in this species against the oomycete pathogen *P*. *infestans* as quantitative resistance which is largely determined by host genotypes.

In our previous work, we found significant differences in resistance of the different *S*. *chilense* populations but also had some very important remaining questions (Stam, Silva‐Arias, et al., 2019). We observed a large amount of variation in the infection success rate for each of the populations—in some populations ranging from 0/10 infections on one leaf to 10/10 on another leaf. This left the question of whether infection frequency is generally variable and spore germination of *P*. *infestans* on *S*. *chilense* is strongly affected by stochastic effects. Using a different *P*. *infestans* isolate as in our previous study and studying up to 1,588 of infection events per population, we again observed a high amount of variance within the populations, but also significant differences between the populations (Figure 1) that correlated with our previous results (Stam, Silva‐Arias, et al., 2019). The variance was reduced when looking into individual plants per population (Figure 2), which suggests that the resistance outcome is at least in part dependent on the plant genotype under consideration. One possible explanation for the observed variance between plants is the age and morphology of the used leaves, as older, thicker leaves might be harder to penetrate by the pathogen. The effect of plant age on potato leaflets has been highlighted and shown to be variable among flowering plants or nonflowering plants (Stewart, 1990). We tried to minimize this effect by regularly cutting back our plants and randomly selecting leaves for infection, but it remains possible that the selected leaves from some plants were less uniform than for other plants, because *S*. *chilense* is known to be a morphologically diverse species, with large variation between and within populations (Raduski & Igić, 2020) and many quantitative resistance phenomena have been described to be age and physiology‐dependent (Niks et al., 2015). We also observed high variability of infection frequency on individual leaves from the same population or even from the same plant. This may have limited the number of statistical significant differences between our genotypes. However, due to the high number of repetitions, we consider these results robust and valuable. They reflect not just a single developmental stage or specific leaf level as considered in most model plant experiments but rather the spectrum of pathogen resistance responses in each given plant. Infections of wild and cultivated tomato with the pathogen *A*. *solani* also often resulted in no visible lesions on some of the leaves of generally susceptible accessions (Chaerani et al., 2007).

That within individual plants the variance observed was still high indicates other effects, such as stochastic effects affecting spore germination rates. Variation in spore germination rate is commonly observed with *P*. *infestans*, also under conductive laboratory conditions (Minogue & Fry, 1981). However, with the high spore concentrations used in our experiments, such variation should not have a strong effect on the outcome of our results.

Another limitation of our previous study was that we tested only a single isolate of *P*. *infestans*. It is well known that *P*. *infestans* strains are genetically diverse, with complete presence–absence polymorphisms of certain key virulence effector genes (Thilliez et al., 2019). Also, different isolates of *P*. *infestans* have been shown to be tremendously different in their aggressiveness on a single host (Cooke et al., 2012). Testing a single isolate prevented drawing general conclusions on the observed resistance in *S*. *chilense*, as outcomes could have been isolate specific.

To address this, we looked into the infection frequency of the central population (LA3111) upon inoculation with seven different isolates of *P*. *infestans*. The infection frequency for most resistant and least resistant plant showed similar outcome regardless of the *P*. *infestans* isolate used (Figure 4). Looking into the growth phenotype of these isolates on culture medium (Figure 3) and correlating it to infection frequency on plant, it becomes evident that there is no correlation in infection frequency on plants and radial growth on plates. We further confirmed the effect of plant genotype to be higher than the pathogen isolate effect on the infection frequency with GLMM. Multiway ANOVA showed no significant role of interactions between plant genotype and pathogen isolate in the resistance outcome indicating that the observed quantitative resistance in our study is not the result of mutual adaptation. Thus, the differences observed in our infection assays are more clearly defined by the plant genotype than the pathogen isolate although we observed an influence of pathogen isolate (Tables 2 and 3). This does not need to depend on isolate germination and growth rate on agar plates, but can be related to various other factors that contribute to the general aggressiveness of individual isolates (Pariaud et al., 2009).

Even though we used seven different isolates, from different hosts, we used only one Latin American isolate from a clonal lineage that is spreading throughout Peru (Lindqvist‐Kreuze et al., 2020). Therefore, there is a possibility that adapted pathogens (e.g., collected directly from *S*. *chilense*) would show different results, which would be interesting to explore in future. As shown by Thrall and Burdon (2003), in more resistant flax populations more virulent rust was observed. This mechanism is also shown in resistant plants of *Plantago* *lanceolata* and powdery mildew *Podosphaera plantaginis* (Laine, 2005). However, field observations by Lindqvist‐Kreuze et al. 2020 and our own observations from field visits in Peru and Chile suggest that in nature, *S*. *chilense* rarely shows strong visible infection with *P*. *infestans* and the pathogen was to our knowledge never successfully isolated from plants from southern populations. Thus, we expect relatively little influence of specific local adaptations and hypothesize that our results represent general defense differences that are potentially functional against a wide array of pathogens. The absence of strong signatures of local adaptation to a pathogen is also shown in case of 12 species of wild potatoes against 13 populations of the nematode *Globodera* *pallia*. Similar as in our study, host genotype was the deciding factor in resistance rather than the pathogen isolate under consideration (Gautier et al., 2020). Such basal resistances have been shown to be prevalent in the plant kingdom and are expected to be based on more conserved, or core, defense mechanisms, like the recognition of microbe‐/pathogen‐associated molecular patterns (M/PAMPS). The presence of basal resistance, which is sometimes also described as exapted resistance (e.g., resistance that is present in a plant, without recent adaptation to the pathogen: Newcombe, 1998), has been studied in the context of invasive *Phytophthora* species on several hosts. In case of Norway spruce, *Picea* *abies*, and Nordmann fir, *Abies* *nordmanniana*, in Swedish Christmas tree farms, which currently are not under threat by *Phytophthora*, it has been shown that after inoculation with different isolates of *Phytophthora* sp. (isolated from water and soil samples from the same region), all the seedlings of these plants showed a basal level of resistance under laboratory conditions (Pettersson et al., 2019). A study by Redondo et al. (2020) on 72 trees of black alder, *Alnus glutinosa* from 12 different sites, showed that all the plants possess a basal level of resistance against *Phytophthora uniformis* before invasion by the pathogen, but also that seedlings from pathogen‐invaded locations were more resistant than seedlings from uninvaded locations. Exapted resistance is also shown in the case of powdery mildew, *Erysiphe alphitoides*, and *Phytophthora cinnamomi* in European oak (*Quercus* *rubur*) where multiple genes were shown to be responsible for the variation in resistance between the trees of naive, not previously infected, populations (Bartholomé et al., 2020).

The molecular mechanisms underlying the observed phenotypes in our study remain thus far unknown. We did not observe any host genotype–pathogen isolate combination that led to complete resistance or showed hypersensitive reactions, suggesting that no major *R* genes against the used isolates are active in those populations. It has been shown that the perception of conserved M/PAMPs can differ within and between wild tomato species (Roberts et al., 2019). The mechanisms of resistance can also vary within a species as in case of natural accessions of *Arabidopsis thaliana* where few accessions showed resistant against the bacterial pathogen *Pseudomonas* *syringae* pv. tomato (*Pst*) DC3000. Resistance mechanisms, such as elevated phytohormone levels or the presence of leaf surface barriers, were shown to differ between the accessions (Velásquez et al., 2017). Differences in defense activation within the species are also shown against a generalist herbivore African cotton leafworm, *Spodoptera littoralis*, on the host plant Arugula, *Eruca* *sativa*, where the activated defense mechanism was associated with geographical origin of the population (Ogran et al., 2016).

In a study with a panel of six domesticated and six wild tomato accessions, infection severity of 97 *Botrytis cinerea* isolates was shown to be determined by complexity of both host and pathogen genotype and a few pathogen genes associated with host preferences (Soltis et al., 2019). Similar multigenic effects has been indicated in a genome‐wide association mapping analysis of 88 genotypes of quinoa, *Chenopodium quinoa*, showing large variation in disease traits upon inoculation with one isolate of the downy mildew pathogen *Peronospora variabilis* (Colque‐Little et al., 2021). With the recent availability of a reference genome sequence for *S*. *chilense* (Stam, et al., 2019), studies to identify the multiple genes underlying the observed differences in quantitative resistance will be the next step.

Our findings show an example of intraspecific variation in quantitative resistance in *S*. *chilense* plants from selected populations, in which we earlier reported variation in qualitative resistance against another pathogen (Kahlon et al., 2020). We show that this quantitative variation exists between and within the natural populations of the species and is mainly driven by the plant genotype. The resistance can potentially be defined as exapted resistance as it appears to protect against nonlocal, un‐adapted pathogen isolates. Overall, we show that the plants of the wild tomato species *S*. *chilense* have their own unique resistance and that the species is amenable for detailed studies on quantitative resistance in the laboratory. In future studies, it would also be interesting to look into structural and molecular factors underlying these quantitative differences and possibly associate this with genetic differences.

## CONFLICT OF INTEREST

The authors declare that no competing interests exist.

## AUTHOR CONTRIBUTIONS

**Parvinderdeep S. Kahlon:** Conceptualization (equal); Data curation (lead); Investigation (lead); Methodology (equal); Visualization (equal); Writing‐original draft (lead); Writing‐review & editing (equal). **Melissa** **Verin:** Formal analysis (supporting); Investigation (supporting); Writing‐review & editing (supporting). **Ralph** **Hückelhoven:** Conceptualization (supporting); Supervision (supporting); Writing‐review & editing (supporting). **Remco Stam:** Conceptualization (lead); Data curation (supporting); Formal analysis (supporting); Methodology (lead); Project administration (lead); Supervision (equal); Writing‐original draft (supporting); Writing‐review & editing (equal).

### OPEN RESEARCH BADGES

This article has been awarded Open Data, Open Materials Badges. All materials and data are publicly accessible via the Open Science Framework at https://github.com/remco‐stam/KahlonEcoEvol.

## Supporting information

Tables S1–S8

Figure S1

Figure S2

Figure S3

Figure S4

## Data Availability

Supplemental tables are made available as supporting information. Raw Data and R scripts are available on github (https://github.com/remco‐stam/KahlonEcoEvol).
