# Supplementary figures and images for: Quantitative resistance differences between and within natural populations of Solanum chilense against the oomycete pathogen Phytophthora infestans
Source: Ecol Evol. 2021 May 11;11(12):7768–78. doi: 10.1002/ece3.7610 (PMC8216925; doi:10.1002/ece3.7610)

a

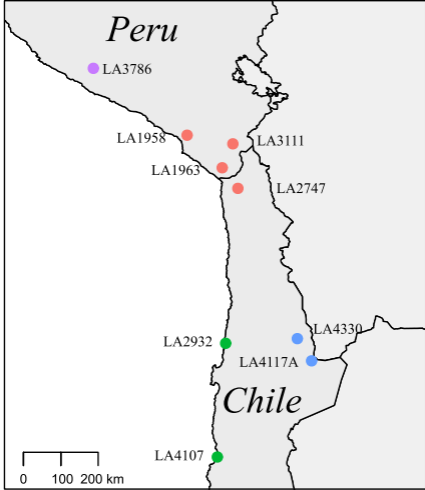

b

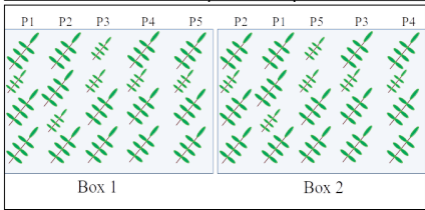

Supplement: Supplementary file 2 — Figure S1 [file ECE3-11-7768-s005.pdf]

Non-sterile  
explant

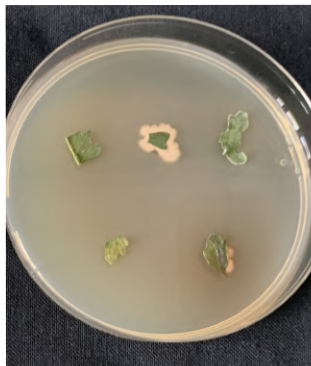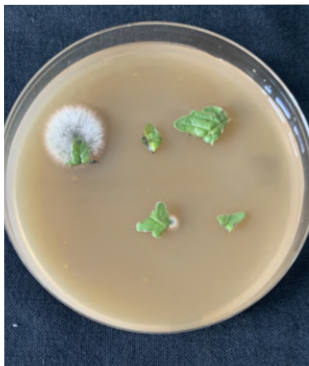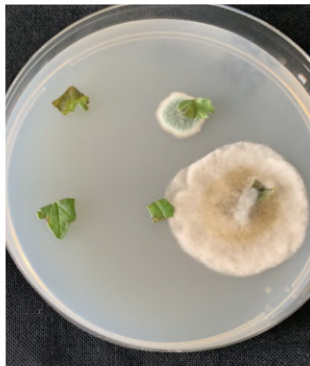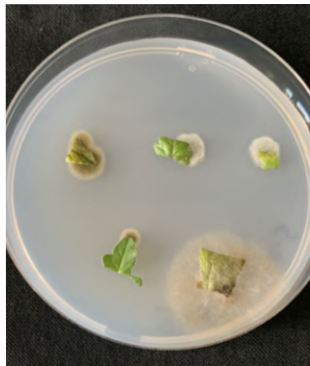

Sterile  
explant

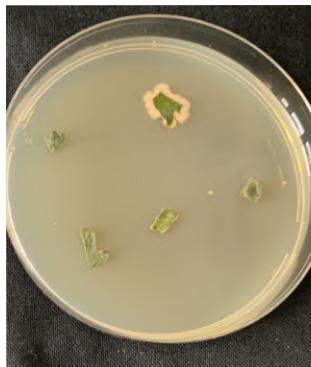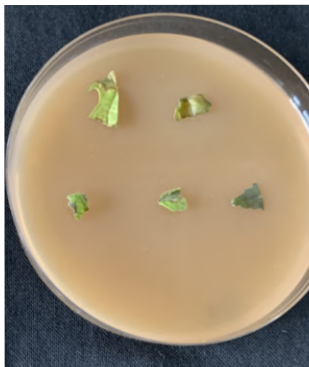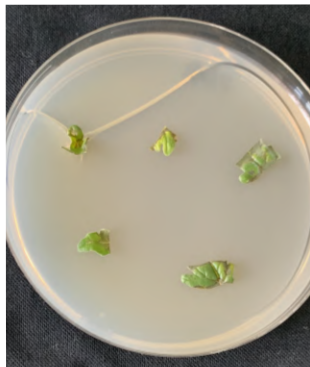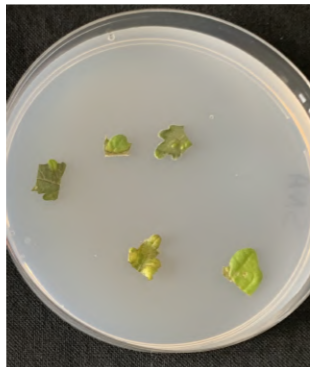

LBA

V8

PDA

SNA

Supplement: Supplementary file 3 — Figure S2 [file ECE3-11-7768-s003.pdf]

Pi100

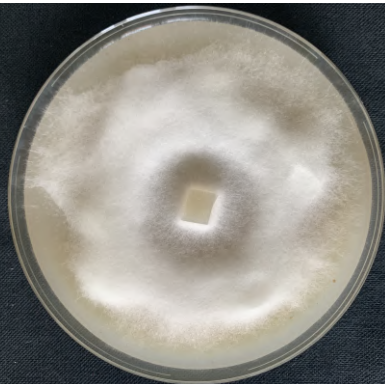

Pi054

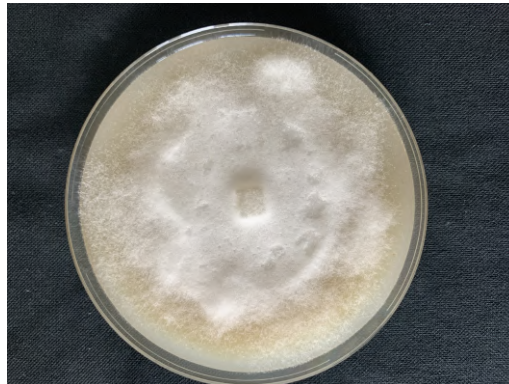

Pi078

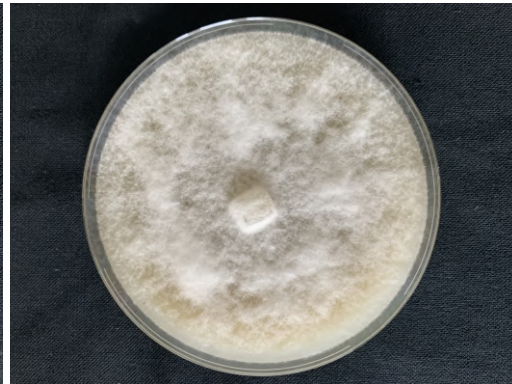

06\_3928A

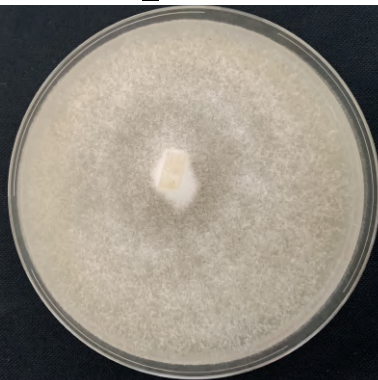

NL88069

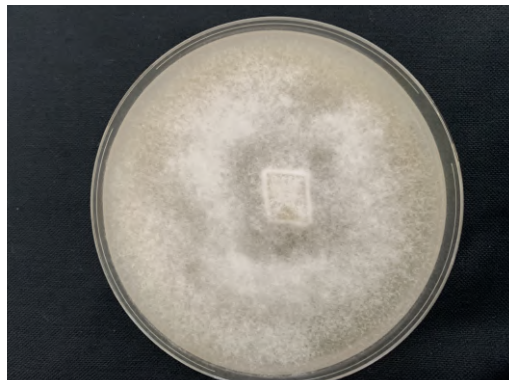

EC1

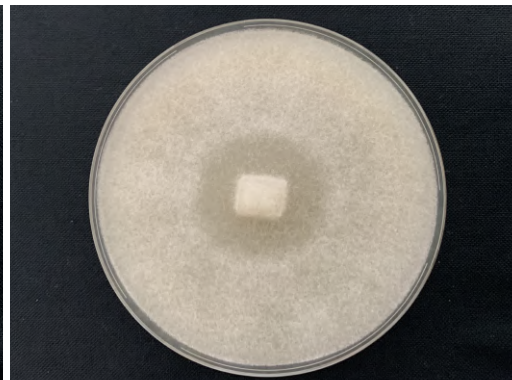

T30-4

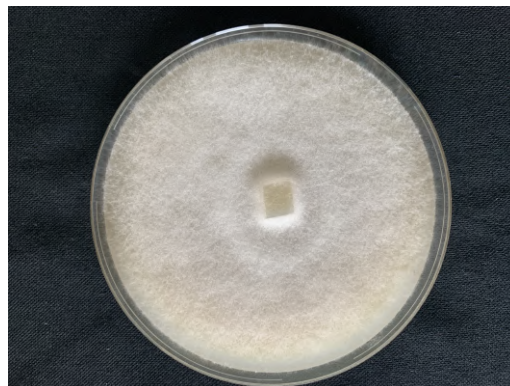

Supplement: Supplementary file 5 — Figure S4 [file ECE3-11-7768-s002.pdf]
