# Supplementary material for: Quantitative resistance differences between and within natural populations of Solanum chilense against the oomycete pathogen Phytophthora infestans
Source: Ecol Evol. 2021 May 11;11(12):7768–78. doi: 10.1002/ece3.7610 (PMC8216925; doi:10.1002/ece3.7610)
Supplement: Supplementary file 4 — Figure S3 [file ECE3-11-7768-s001.pdf]

a) Examples of control (water) inoculated leaves (7 days post inoculation)

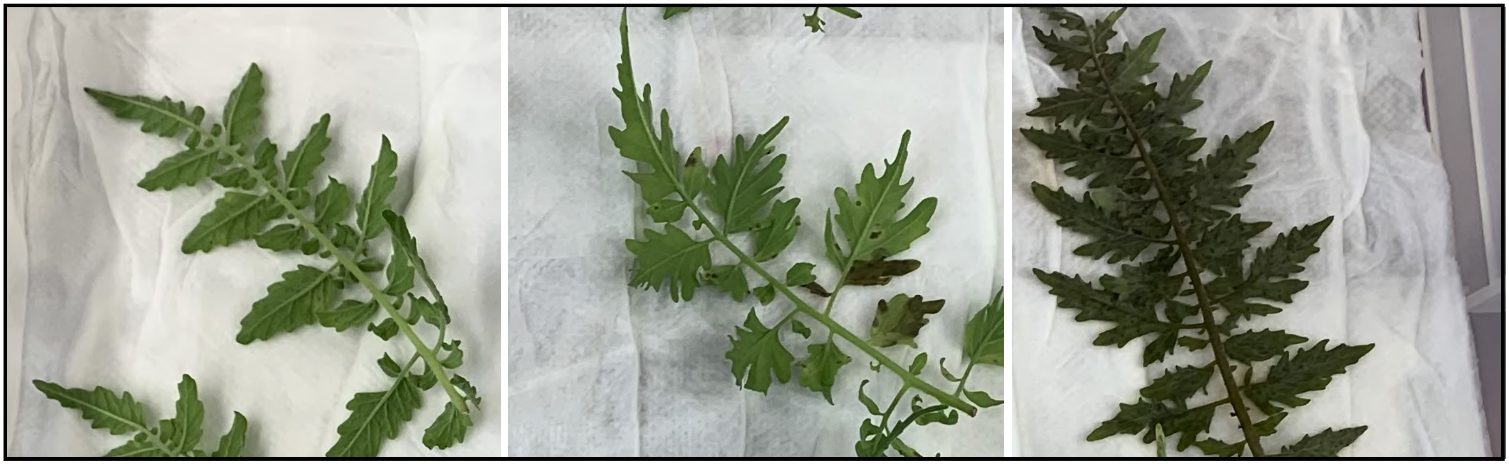

No contamination | Minor contamination | Major contamination

b) Examples of inoculation output considered in the data analysis

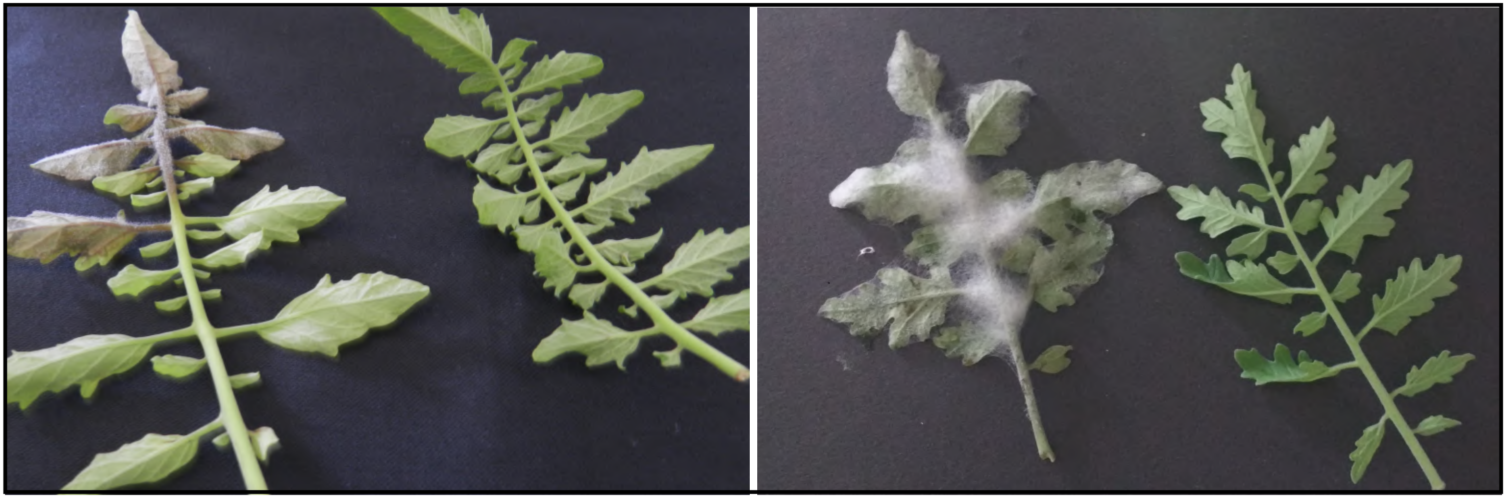

P. infestans inoculated | Water inoculated | *P. infestans* inoculated | Water inoculated
